# Immune Response After Rabies Vaccination in Owned Free-Roaming Domestic Dogs in Flores Island, Indonesia

**DOI:** 10.3389/fvets.2022.868380

**Published:** 2022-06-09

**Authors:** Ewaldus Wera, Charlotte Warembourg, Petrus M. Bulu, Maria M. Siko, Salome Dürr

**Affiliations:** ^1^Animal Health Study Program, Kupang State Agricultural Polytechnic (Politeknik Pertanian Negeri Kupang), Kupang, Indonesia; ^2^Vetsuisse Faculty, Veterinary Public Health Institute, University of Bern, Bern, Switzerland; ^3^Animal Health Division, Agricultural Department of Sikka Regency, Maumere, Indonesia

**Keywords:** rabies antibody, free roaming dogs, vaccine, Flores Island (Indonesia), body condition score (BCS), history of vaccination, serology

## Abstract

Vaccination is the main tool to prevent the circulation of rabies in dog populations. The development of an immune response after vaccination differs between individual dogs and depends on many factors such as dog characteristics, management, or genetics. Here, we first investigated the level of, and associated factors for, the presence of binding antibodies in 130 healthy dogs from Flores Island, Indonesia. Secondly, we identified factors associated with the development of binding antibodies within 30 days after vaccination among a subsample of dogs that had a binding antibody titre <0.5 EU/ml at the day of vaccination (D0, *N* = 91). Blood samples were collected from the individual dogs immediately before vaccination at D0 and 30 days after vaccination (D30). The rabies antibody titres were determined using ELISAs. Information on potential risk factors such as the dog's age and sex, history of vaccination, type and frequency of feeding, and BCS (body condition score) were gathered during interviews at D0. Regression analyses were performed to identify the risk factors associated with the presence of binding antibody titre ≥0.5 EU/ml at D0 for the 130 dogs and the development of binding antibody titre ≥0.5EU/ml at D30 for the 91 dogs. The results showed that the proportion of dogs with antibody titre ≥0.5 EU/ml was 30% (39/130) at D0. The only factors found to be significantly influencing the presence of binding antibodies titres ≥0.5 EU/ml was previous vaccination within 1 year before D0 [46.8 vs. 14.7%, Odds ratio (OR) = 3.6, 95%CI 1.5–9.3; *p*-value = 0.006], although the same trend was found for dogs of higher age and better BCS. Eighty-six percent (79/91) of dogs whose rabies binding antibody level was <0.5 EU/ml at D0 had developed an adequate immune response (≥0.5 EU/ml) at D30. Almost a significantly higher proportion developed an adequate immune response in dogs of good BCS compared to those of poor BCS (95.3% vs. 79.2%, OR = 4.7, 95%CI 1.1–32.5; *p*-value = 0.057. Twelve (13.2%) dogs retain binding antibody level <0.5 EU/ml at D30, indicating poor immune response after vaccination. A majority of them did not receive vaccine before D0 according to the owner and had poor BCS (83.3%; 10/12). Our findings show the high effectiveness of rabies vaccine in under field conditions to develop measurable immunity and the importance of a good BCS, often achievable by good dog keeping conditions, for developing efficient immunity after parenteral vaccination in dogs.

## Introduction

Rabies remains an important global health concern, with an estimated global annual human death of 25 to 159 thousand cases (1). Most of these cases occur in African and Asian developing countries, with 60% of the global disability adjusted live years lost due to rabies occurring in Asia (1). Human rabies cases in developing countries are mostly transmitted by domestic dogs. Although they are mainly kept free-roaming, dogs in developing countries have a function, as for example in Indonesia where they are often kept as guardians for properties and to chase away wild animals, particularly monkeys and wild pigs (2, 3). Vaccination of dogs has been repeatedly shown to be the most effective strategy to control rabies in a sustainable way. However, diverse challenges, from political to organisational, have been identified to reach a high enough vaccination coverage in dog populations (4). As a consequence, the annual vaccination coverage is most often lower than the vaccination coverage of 70% recommended by WHO for preventing the circulation of rabies virus among dog population, as for example 53% that was reached in Flores Island, Indonesia over the period 2000–2011 (5).

In addition to a high coverage of mass dog vaccination, providing high-quality dog vaccine is key for the success of canine rabies elimination programs (6). High quality and effective rabies vaccine exist on the market, with laboratory trials documenting 93% seroconversion in vaccinated dogs after a single dose (7). However, development of immunity under field conditions might be hampered by several factors. Identifying them, helps to inform effective rabies vaccination campaigns.

Several studies have been conducted in European countries to identify risk factors of development of antibodies in dogs after vaccination (8–13). Age and breed are amongst the most commonly identified factors correlated with the lack of detectable immunity production after vaccination in dog population (9–12). For example, Keneddy et al. studied the antibody response of 10,483 dogs tested at the Veterinary laboratories Agency, Weybridge UK and found that adult dogs aged 1–7 years were more likely to have an antibody titre >0.5 International Units per ml serum (IU/ml) than young dogs (<1 year) (9). Berndtsson et al. investigated factors associated with the development of antibody titre of 6,789 vaccinated dogs in Sweden and found that larger breeds were at higher risk of having antibody titre <0.5 IU/ml (12). However, most of the above studies were conducted in developed countries for the purpose of international pet travel. Field studies to identify risk factors of development of antibodies in free-roaming domestic dogs in developing countries are limited (14, 15). In a randomised controlled study conducted in Tanzanian dogs, Lugelo et al. found that there was a positive correlation between body condition score (BCS) and seroconversion in which those dogs with good BCS were more likely to seroconvert than dogs with poor BCS (14). In contrast, Morters et al. investigated risk factors of antibody response of the free-roaming domestic dogs after vaccination in South Africa and found that the vast majority of the dogs seroconverted 30 days after vaccination, regardless of the health status of vaccinated dogs (15).

The majority of free-roaming dogs in Indonesia, as in most of developing countries, are aged <1 year and have a poor BCS as a consequence of insufficient nutrition uptake or parasitic diseases (16). The influence of BCS on the losing antibody titre has been reported in the previous studies (17). In a prospective study, Wera et al. found that the dogs with low BCS tend to more rapidly lose detectable antibody titres after vaccination than dogs with high BCS (17). However, to which extent the dogs develop binding antibodies after vaccination has not yet been analysed in these populations. In this study, we first investigated the level of and associated factors for the presence of binding antibodies in 130 healthy owned free roaming dogs from Flores Island, Indonesia. Some of them have previously been vaccinated, and the vaccination status was based on owners' reports. This study approach was previously used in the literature (10, 12). However, the low level of antibodies in the investigated dogs does not fully represent the development of immunity, because dogs may have already lost detectable binding antibodies after vaccination, although cellular immunity may still be present (18, 19). Therefore, we secondly investigated binding antibody titres and identified factors associated with the development of antibodies within 30 days after vaccination among a subsample of dogs that had a binding antibody titre <0.5 Equivalent Unit per ml serum (EU/ml) at the day of vaccination.

## Materials and Methods

### Study Site and Data Collection

Study site and data collection has been described in a study previously conducted by the authors (17). In brief, blood sampling and questionnaire surveys were conducted in dog-owning households in two rural and one urban area in Sikka Regency, Flores Island, Indonesia between July and September 2018. As the dog sampling was part of a larger project on the investigation of dogs' roaming behaviour (3, 16), research cohort included every available healthy dog aged 3 months and more in the study area of 1 km^2^ and excluded sick dogs. In addition, pregnant dogs were excluded from the study to avoid miscarriage due to stress. Dog blood samples were collected from 256 dogs on the day of vaccination (D0) and each dog was vaccinated subcutaneously with 0.5 ml of OIE pre-qualified vaccine (Rabisin vaccine, Boehringer Ingelheim) immediately after the blood sampling. Blood was further sampled from the exact same dogs 30 days after vaccination (D30), in case they were available. Information on dog characteristics, history of vaccination (previous vaccination performed and date of vaccination), kind of daily food (leftovers, rice, corn, and fish), frequency of feeding, and BCS was collected during interviews. The history of vaccination in the present study was mainly based on the dog owners' declaration. The number of dogs feeding with corn and fish is too low (nine dogs) to be analysed, therefore, rice, corn, and fish were collapsed into “others.” The investigators were equipped with the pictures of BCS categorisation according to The American Animal Hospital Association (20) at D0. The BCS was assigned by the investigators (based on the pictures) using a 5-point system and categorised as “too thin” (BCS 1), “thin” (BCS 2), “ideal” (BCS 3), “overweight” (BCS 4), and “obese” (BCS 5). For purpose of data analysis, categories were collapsed as “good” (BCS 3, 4, 5) and “poor” (BCS 1 and 2) BCS. The interviews were conducted in Bahasa by the research team.

Of 256 blood samples collected at D0, 126 (49.2%) blood samples showed lysis due inappropriate handling after blood collection and had to be excluded from laboratory analysis. Consequently, 130 samples were eligible to be tested for the presence of rabies antibodies. For the first analysis, the 130 dogs with a serology result at D0 were investigated. For the second analysis, as the aim of the study was to identify the development of binding antibody titres within 30 days after vaccination and risk factors associated with binding antibody development, the analysis was conducted on the 91 dogs that manifested an antibody titre <0.5 EU/ml at D0.

At the same day of sampling, full blood was centrifuged and the serum was extracted, which was then dispensed into 3 ml labelled Eppendorf tubes. The tubes were stored at +4°C until shipment to the veterinary laboratory (Disease Investigation Centre, Denpasar) in Bali. The presence of rabies antibodies of the serum samples was tested by ELISA as described elsewhere (15). Samples were categorised as negative, i.e., having inadequate level of binding antibodies, if the titre was detected to be <0.5 EU/ml, and positive otherwise.

### Statistical Analysis

Categorical variables were presented as numbers with their corresponding percentages. Univariable and multivariable logistic regression analyses were conducted to investigate the effect of the independent variables (Table 1) on the presence (D0 model) and development (D30 model) of binding antibodies after vaccination (outcome variable). All independent variables that had *p*-values lower than or equal to 0.25 in the univariable analyses were subsequently included in the initial models for the multivariable analyses (21). The final multivariable logistic models were derived by comparing the Akaike Information Criterion (AIC) of the models build with all possible combinations of the variables selected for the multivariable analysis. The model with the lowest AIC was considered as the final model. The fit of the final models to the data was determined using the Hosmer-Lemeshow goodness-of-fit test (21). The final models were considered a good fit for the data if the *p*-value of the Hosmer-Lemeshow test was greater than 0.05. All statistical analyses were performed with SPSS version 19 and R version 4.1.0 (function *glm* of the *stats* package for the regression analysis and function *hoslem.test* of the package *Resource Selection* for the Hosmer-Lemeshow test). We assumed a level of significance at 0.05.

**Table 1:** Demographic characteristics of dogs surveyed in Flores Island, Indonesia on the days of vaccination, D0 (*n* = 130) and at 30 days after vaccination, D30 (*n* = 91; only dogs with antibody titres <0.5 EU/ml at D0 were considered for the analysis).

**Variable**	**D0^a^ (*****N*** **= 130 dogs)**		**D30 (*****N*** **= 91)**	
	**Frequency (*n*)**	**Percentage (%)**	**Frequency (*n*)**	**Percentage (%)**
Sex				
Female	88	67.7	59	64.8
Male	42	32.3	32	35.2
Age				
<12 months	74	56.9	60	65.9
≥12 months	56	43.1	31	34.1
Breed				
Local breed	126	96.9	88	96.7
Other	4	3.1	3	3.3
Geographical area				
Urban	69	53.1	50	54.9
Rural	61	46.9	41	45.1
History of rabies vaccination				
Vaccinated <12 months before D0^a^	62	47.7	39	40.7
Never vaccinated or vaccinated >12 months before D0^a^	68	52.3	52	59.3
Origin of dogs				
Born at home	54	41.5	38	40.7
Given or bought	76	58.5	53	59.3
Kind of daily food				
Leftovers	121	93.1	83	90.1
Other^b^	9	6.9	8	9.9
Frequency of feeding				
<3 times per day	76	58.5	52	57.1
≥3 times per day	54	41.5	39	42.9
Body condition score /BCS^c^				
Poor	66	50.8	48	52.7
Good	64	49.2	43	47.3

a *D0 is the day of vaccination within this study*.

b *Other daily food like rice, corn, fish*.

c *BCS ranged from 1 to 5 and was categorised as poor for scores lower than 3 and good for scores of 3 or higher*.

## Results

### Study Population

A total of 130 free-roaming owned dogs from 98 households in two rural (Pogon and Hepang) and one urban (Habi) area were eligible to be tested for antibody against rabies at D0 (Table 1). The majority of dogs were younger than 12 months (56.9%), had a poor BCS of <3 (50.8%), and had either no previous vaccination or was vaccinated more than 12 months before D0 (52.3%). These characteristics were comparable with the characteristics of the 91 dogs sampled and included in the analysis at D30 (Table 1).

### Antibody Titres and Factors Influencing the Presence of Adequate Binding Antibodies Levels at D0

At D0, 39 of the 130 dogs (30.0%) had antibody titres ≥0.5 EU/ml. A higher proportion of female dogs had antibody titre ≥0.5 EU/ml compared to male dogs (Table 2). Similarly, the proportion of rural dogs with antibody titre ≥0.5 EU/ml was higher than urban dogs, but the differences were not significant in the univariable analysis (*p* > 0.05) (Table 2). Furthermore, of the 62 dogs that had a history of vaccination within 12 months before D0, 29 (46.8%) had antibody titres ≥0.5 EU/ml against rabies. Only 10 (14.7%) of the 68 dogs with either no previous vaccination or were vaccinated more than 12 months before D0, had antibody titres ≥0.5 EU/ml, which was significantly less than dogs vaccinated within the last 12 months before D0 (Table 2). Dogs older than or equal to 12 months were significantly more likely to have antibody titres more than ≥0.5 EU/ml (18.9 vs. 44.6%, *p* = 0.002) than those age <12 months. In the multivariable analyses, the history of vaccination was the only factor significantly associated with the proportion of binding antibody titres ≥0.5 EU/ml at D0 (Table 3). Being of age more than 12 months and having a good BCS also increased the odds of antibody titres being >0.5 EU/ml, however not on a significant level according to our defined significance level.

**Table 2:** Frequency (*n*) and percentage (*n*/*N*) of dogs having a level of ≥0.5 EU/ml of binding antibodies at D0, stratified by different demographic characteristics of the dogs.

**Variables**	**Sample**	**Binding antibody titre**	**Percentage**	**OR**	**95% CI of OR**	***P*** -value^*^
	**size (*N*)**	**≥0.5 EU/ml (*n*)**	**(*n*/*N* in %)**			
Sex						0.287
Male	42	10	23.8	1.00		
Female	88	29	33.0	1.57	0.68–3.64	
Age						**0.002**
<12 months	74	14	18.9	1.00		
≥12 months	56	25	44.6	3.46	1.58–7.58	
Breed						1.000
Local breed	126	38	30.2	1.30	0.13–12.86	
Other	4	1	25.0	1.00		
Geographical area						0.541
Urban	69	19	27.5	1.00		
Rural	61	20	32.8	1.28	0.61–2.72	
History of rabies vaccination						**<0.001**
Vaccinated <12 months before D0^a^	62	29	46.8	5.10	2.21–11.76	
Never vaccinated or vaccinated >12 months before D0^a^	68	10	14.7	1.00		
Origin of dogs						0.938
Born at home	54	16	29.6	1.00		
Given or bought	76	23	30.3	1.03	0.48–2.21	
Kind of daily food						0.277
Leftovers	121	38	30.0	3.66	0.44–30.33	
Other^b^	9	1	11.1	1.00		
Frequency of food						0.641
<3 times per day	76	24	31.6	1.20	0.56–2.58	
≥3 times per day	54	15	27.8	1.00		
Body condition score^c^						**0.026**
Poor	66	14	21.2	1.00		
Good	64	25	39.1	2.38	1.10–5.17	

*The influence of demographic parameters was explored by univariable logistic regression analyses*.

*OR, Odds ratio; CI, Confidence interval*.

a *D0 is the day of vaccination in this study*.

b *Other daily food include rice, corn, or fish*.

c *BCS ranged from 1 to 5 and was categorised as poor for scores lower than 3 and good for scores of 3 or higher*.

* *p-value shown in bold represents p ≤ 0.25; these variables were used in the subsequent multivariable logistic regression analysis*.

**Table 3:** Determinants associated with developing of adequate level of binding antibodies at D0 in dogs on Flores Island, Indonesia, using multivariable logistic regression analysis.

**Variable**	***n*** **/** ***N***	**percentage**	**OR (95% CI)**	***p*** **-value**
History of rabies vaccination before D0^*^				0.006
≥12 months	10/68	14.7	1.00	
<12 months	29/62	46.8	3.63 (1.4–9.30)	
Age				0.141
<12 months	14/74	18.9	1.00	
≥12 months	25/56	44.6	1.94 (0.80–4.70)	
Body condition score				0.127
Poor	14/66	21.2	1.00	
Good	25/64	39.1	1.91 (0.84–4.44)	

*OR, Odds ratio; CI, Confidence interval*.

* *D0 is the day of vaccination within this study*.

### Antibody Titres and Factors Influencing the Presence of Adequate Binding Antibodies Levels at D30

At D30, 79 (86.8%) of the 91 dogs with detectable levels of rabies antibody titres <0.5 EU/ml at D0 developed antibody titres ≥0.5 EU/ml. No significant differences were found for factors age, sex, breed, geographical area of dogs (urban vs. rural), kind of daily food, and frequency of feeding (Table 4). Univariable analyses showed that almost significantly (OR = 4.4, 95%CI: 0.9–21.4; *P* = 0.066) more dogs (37/39, 94.9%) with previous vaccination within 12 months before D0 developed antibody titres ≥0.5 EU/ml at D30, compared to 42 (80.8%) of the 52 dogs without history vaccination within 12 months before D0 (Table 4). Forty-one (95.3%) of 43 dogs with good BCS had antibody titres ≥0.5 EU/ml at D30, which was significantly more than the 79.2% among dogs with poor BCS. Of the 12 (13.2%) dogs that had an inadequate immune response at D30 (i.e., binding antibody level <0.5 EU/ml), 10 dogs (83%; 10/12) did not receive vaccines within 12 months before D0 and had poor BCS, while the other two dogs had vaccination within 12 months before D0, but had a poor BCS.

**Table 4:** Frequency (*n*) and percentage (*n*/*N*) of dogs developing a level ≥0.5 EU/ml of binding antibodies within 30 days after vaccination, stratified by different demographic characteristics of the dogs.

**Variable**	**Sample**	**Binding antibody titre**	**Percentage**	**OR**	**95% CI of OR**	***P*** -value^*^
	**(*N*)**	**≥0.5 EU/ml (*n*)**	**(*n*/*N* in %)**			
Sex						0.747
Male	32	27	84.4	1.00		
Female	59	52	88.1	0.73	0.21–2.51	
Age						0.745
<12 months	60	51	85.0	1.00		
≥12 months	31	28	90.3	1.65	0.41–6.58	
Breed						1.000
Local breed	88	76	86.4	NA		
Other	3	3	100			
Geographical area						0.800
Urban	50	43	86.0	1.00		
Rural	41	36	87.8	1.17	0.34–4.01	
History of rabies vaccination						**0.066**
Vaccinated <12 months before D0^a^	39	37	94.9	4.41	0.91–21.41	
Never vaccinated or vaccinated >12 months before D0^a^	52	42	80.8	1.00		
Origin of dogs						0.525
Born in house	38	34	89.5	1.00		
Given or bought	53	45	84.9	0.66	0.18–2.38	
Kind of daily food						0.301
Leftovers	83	73	88.0	2.43	0.43–13.75	
Other^b^	8	6	75.0	1.00		
Frequency of food						0.929
<3 times per day	52	45	86.5	0.94	0.28–3.24	
≥3 times per day	39	34	87.2	1.00		
Body condition score^c^						**0.023**
Poor	48	38	79.2	1.00		
Good	43	41	95.3	5.39	1.11–26.22	

*The influence of demographic parameters was explored by univariable logistic regression analyses*.

a *D0 is the day of vaccination within this study*.

b *Other daily food like rice, corn, fish*.

c *BCS ranged from 1 to 5 and was categorised as poor for scores lower than 3 and good for scores of 3 or higher*.

* *p-value shown in bold represents p ≤ 0.25; these variables were used in the subsequent multivariable logistic regression analysis*.

Multivariable analyses showed that BCS was almost significantly associated with the development of binding antibody levels of >0.5 EU/ml at D30 with almost a significantly higher proportion of adequate immune response found in dogs with good BCS compared to those poor BCS (95.3 vs. 79.2%, *p*-value = 0.057) (Table 5). Dogs being vaccinated <12 months before D0 also tended to have a higher odd of developing an antibody titre of >0.5 EU/ml, however the effect was observed to be lower than for the BCS (Table 5). In both multivariable regression models (Tables 3, 5), the Hosmer-Lemeshow goodness-of-fit test *p*-values were non-significant (*p* = 0.982, *p* = 0.999 for model D0 and D30, respectively), indicating the model fitted the data well.

**Table 5:** Determinants associated with developing of adequate level of binding antibodies 30 days after rabies vaccination in dogs on Flores Island, Indonesia, using multivariable logistic regression analysis.

**Variable**	***n*** **/** ***N***	**percentage**	**OR (95% CI)**	***p*** **-value**
Body condition score (BCS)^*^				0.057
Poor	38/48	79.2	1.00	
Good	41/43	95.3	4.72 (1.12–32.45)	
History of rabies vaccination before D0^*^				0.107
≥12 months	42/52	80.8	1.00	
<12 months	37/39	94.9	3.75 (0.08–25.93)	

*OR, Odds ratio; CI, Confidence interval*.

* *BCS ranged from 1 to 5 and was categorised as poor for scores lower than 3 and good for scores of 3 or higher*.

## Discussion

The success of a parenteral mass vaccination campaign in the field depends on many factors. These include dog owners' behaviour, infrastructure, the sustainability of vaccination campaign programs, and immunity coverage after vaccination. A good balance of these factors could contribute to effective prevention of rabies both in dogs and humans. For example, 2 years mass dog vaccination campaigns in Bali using long-acting vaccines with a coverage of >70% in 2010 and 2011 have led to the reduction of dog and human rabies cases by ~90% (5). Similarly, dog and human rabies incidence in Latin America and Caribbean has successfully decreased by 90% due to sustainable efforts in mass dog vaccination campaigns using a high quality of rabies vaccines (22). A high quality of rabies vaccines is expected to guarantee protection in dogs from rabies, resulting in a high immunity coverage. Vaccination coverage of 70% after sustained annual vaccination is necessary to interrupt the rabies virus circulation among dogs and is thus more cost-effective than PEP alone for preventing human rabies (23, 24). In Flores Island, mass dog vaccinations are carried out annually, targeting all dogs older than 3 months, achieving vaccination coverage around 53% on average (5). This low vaccination coverage was unable to eliminate rabies in Flores Island (25, 26). Furthermore, we earlier reported that the loss of detectable immunity is substantial within 1 year after vaccination in dogs on Flores Island, indicating that vaccination campaign on annual basis may be insufficient (17). Currently, the development of binding antibodies after vaccination has not been investigated yet in these populations, which was the objective of this study.

The present study highlighted the proportion of dogs with antibody titre ≥0.5 EU/ml increased from 30% (39/130) at D0 before vaccination to 86.8% (79/91) at D30 (30 days after vaccination). Increasing the proportion of dogs with antibody titre ≥0.5 EU/ml 1 month after vaccination is well-documented in the literature and findings lay in the range of what was found in the present study (7, 14, 27). Lugelo et al. reported that the proportion of dogs having a titre higher than 0.5 IU/ml increased from 4% (D0) to 85% (D30) (14). Similarly, Minke et al. studied the antibody titre in a group of 30 laboratory dogs vaccinated with Rabisin rabies vaccine and found that the proportion of dogs with a titre ≥0.5 IU/ml increased sharply from 0% at D0 to 93% at D30 post-vaccination (7). Furthermore, Kallel et al. studied the immune response of 1,000 vaccinated domestic dogs under field condition in Tunisia and found that the proportion of dogs with a protective antibody titres at D0 and D30 was 30 and 91%, respectively (27). These results indicate that the vaccine used in the present study was as effective as in comparable studies in producing protective antibody against rabies in the field.

Although development of immunity has already been studied in other contexts, it is of interest to investigate the effectiveness of internationally recognised vaccines in field condition, particularly in areas with limited animal health infrastructure and services, like Flores Island. Moreover, the socio-economic status of dog owners differs between countries, leading to varying availability of resources for ideal dog keeping practises. The results of the present study showed that around 13.2% (12/91) of vaccinated dogs have not produced a sufficient level to be protected against rabies despite the use of an international commercialised rabies vaccine well-known for its efficacy in inducing a strong humoral response (7). Failure to produce a strong humoral response 30 days after vaccination (D30) was also previously found in laboratory experiment (7) and under field conditions (14, 28). Although virus challenge studies would be needed to definitively prove the lack of protection against rabies, for the development of immunity, measuring antibody titres are a useful tool (18, 19). In an experimental study, Auber et al. provided evidence that animals with neutralising antibody titres at the time of challenge towards rabies virus were better protected than those without neutralising antibodies (18). Therefore, these 135% of dogs (*n* = 12) are very likely to be not protected at D30, because of their lack of reaction towards the vaccine.

In our study, failure to produce a strong humoral response was linked to low BCS. The multivariable logistic regression analysis results indicated that the main determinant of antibody development at D30 was the BCS. The results are consistent with a field study in Tanzania, in which 412 free-roaming domestic dogs following single dose of rabies vaccination, demonstrated the significant association between BCS and seroconversion (14). Similarly, Wera et al. have reported significantly higher proportion of antibody titres >0.5 EU/ml in dogs with good BCS vs. poor BCS at 90, 180, and 270 days after rabies vaccination (17). These findings suggest that the present study provides consistent evidence for BCS influencing the development and presence of binding antibody titres following rabies vaccination.

This observed association between presence of antibody titres and BCS may be attributed to the health status of dogs at the time of vaccination because other dog characteristics such as age, sex and breed, the location they live (urban vs. rural), and management practises (feeding, origin and role of the dog) did not show any significant relation to the presence of antibody titres. Dogs with poor BCS are malnourished and most frequently found with high burden of endo and ecto-parasites (own unpublished data from West Timor, Indonesia). This is most likely due to the fact that dogs in developing countries are free roaming day and night and therefore encounter more dogs and potentially contaminated environment (2, 15, 16). They have, thus, a high potential to be infected by parasites. This further depresses the immune system and consequently reduce antibody production (29). Booster vaccination focusing on dogs with low BCS and educational campaigns to improve knowledge on dog management practise, promote responsible dog ownership including good feeding and health practises, is expected to improve BCS of the dogs, which would enhance producing immunity after vaccination. We thus argue that investing into improved dog keeping practises leading to an increase of dog health and BCS, could highly improve the efficiency of rabies vaccination campaigns in the future.

In our study, we exclude obviously sick dogs because another part of the project is aimed at investigating normal dog roaming behaviour (3, 16, 30). Investigating the effect of the dog's health status in addition to the effect of BCS on the development of immunity after vaccination in the field should be of interest for future studies.

The present study found that 46.8% of dogs that had a history of vaccination within 12 months before D0 had evidence of anti-rabies antibodies at D0. Having a low level of antibodies (<0.5 EU/ml) does not always indicate poor reaction towards vaccination, because it may be that the dogs have developed immunity after the vaccination in the past, but had a decay in the detectable antibodies until blood sampling. Low coverage of antibody titres >0.5 EU/ml after the mass vaccination against rabies in dog populations was also reported by other authors (27, 31, 32). Tepsumethanon et al. described that 42% of vaccinated dogs in Thailand failed to present rabies antibody titres >0.5 IU/ml 360 days after vaccination (31). Similarly, Cliquet et al. reported low immunity coverage (36%) 1 year after vaccination in a dog population in Tunisia (32). These findings suggest that a large number of dogs are potentially susceptible to rabies 1 year after the vaccination campaign, although cellular immunity that is not measured by the antibodies may still be present (18, 19). It is noteworthy that in our study, the majority of dogs with antibody titres <0.5 EU/ml at D0 are dogs having no history of vaccination before D0 (63.7%; 58/91), which was found to be a significant factor for the D0 titre. Therefore, booster vaccination (for example, 6 months after first vaccination) targeting on dogs without previous vaccination is crucial to increase the immune response between yearly campaign and eventually eliminate rabies both in dogs and humans.

The history of vaccination in the present study was mainly based on the dog owners' declaration. This approach is also reported by other authors (33–35) and may lead to an over- or underestimation of the proportion of vaccinated dogs, as dog owners could misclassify the dog's vaccination status. Dog vaccination campaigns in Flores Island are carried out on a yearly basis by public veterinary offices without providing vaccination certificate for dog owners. Despite this limitation, the results of this study illustrate the link of history of vaccination and the presence of a strong immune response in owned free-roaming domestic dogs in Flores Island. Finally, our study revealed that 14.7% (*n* = 10) of dogs with either no previous vaccination or more than 12 months before D0, had antibody titres ≥0.5 EU/ml at D0. According to the statement of the owners, most of these dogs were previously vaccinated more than 12 months before D0. However, for four (3% of the entire study population of 130 dogs) dogs, owners reported that the dogs were unvaccinated during their life. Although it may be that the statement of the owners was wrong (for example due to lack of memory or change of the dog's owner over time), natural immunity due to exposure to rabies virus not leading to fatal disease can be the reason for this observation (36, 37).

## Conclusion

Our findings showed a high immune response in owned free-roaming domestic dogs vaccinated under field conditions in Indonesia. History of vaccination and good BCS were significantly associated with the presence of rabies antibody at D0 and the development of antibodies at D30. Given the significant association with BCS, an educational campaign focusing on dog management practise is expected to improve the health status and BCS of the dogs, which would enhance producing detectable immunity after vaccination.

## Data Availability Statement

The original contributions presented in the study are included in the article/supplementary material, further inquiries can be directed to the corresponding author/s.

## Ethics Statement

The animal study was reviewed and approved by Animal Ethics Commission of the Veterinary Medicine Faculty, Nusa Cendana University (Protocol KEH/FKH/NPEH/2019/009). Written informed consent for participation was not obtained from the owners because dog owners in Flores were not familiar with written informed consent.

## Author Contributions

EW, CW, SD, PMB, and MMS conceived the study. EW, CW, PMB, and MMS collected the field data. EW, CW, SD, and PMB conducted statistical analyses. EW wrote the original draft of the manuscript. CW, SD, PMB, and MMS contributed to reviewing the manuscript. All authors read and approved the version of the manuscript to be published.

## Funding

The study was funded by the Kupang State Agricultural Polytechnic (Politeknik Pertanian Negeri Kupang) in the form of a grant awarded to EW and PMB (Project Nr. 1/P2M/DIPA.042.01.2.40.1014/2019), the Albert Heim Foundation (Project Nr. 132), and the Wolfermann-Nägeli Foundation (Nr. 2018/28) in the form of a grant awarded to CW and SD.

## Conflict of Interest

The authors declare that the research was conducted in the absence of any commercial or financial relationships that could be construed as a potential conflict of interest.

## Publisher's Note

All claims expressed in this article are solely those of the authors and do not necessarily represent those of their affiliated organizations, or those of the publisher, the editors and the reviewers. Any product that may be evaluated in this article, or claim that may be made by its manufacturer, is not guaranteed or endorsed by the publisher.
